# 

**Published:** 1914-02

**Authors:** 


PUBLISHED MONTHLY. ATLANTA SUBSCRIPTION $1.00

### JOURNAL-RECORD OF MEDICINE

Successor to { Atlanta Medical and Surgical Journal, Established 1855. and Southern Medical Record, Established 1870. } 60th Year

Vol. LX. ATLANTA, GA., FEBRUARY, 1914. No. 11

